# Parent-child communication about sexual and reproductive health in rural Tanzania: Implications for young people's sexual health interventions

**DOI:** 10.1186/1742-4755-7-6

**Published:** 2010-05-12

**Authors:** Joyce Wamoyi, Angela Fenwick, Mark Urassa, Basia Zaba, William Stones

**Affiliations:** 1 National Institute for Medical Research, Mwanza Research Centre, Isamilo Road, P.O Box 1462, Mwanza, Tanzania; 2 University of Southampton, School of Medicine, Division of Medical Education, Boldrewood Campus, Southampton, SO16 7PX, UK; 3 London School of Hygiene and Tropical Medicine, Centre for Population Studies, Keppel Street, London WC1E 7HT, UK; 4 Aga Khan University Hospital, Faculty of Health Sciences, 3 Parklands Avenue, P.O Box 30270-00100-GPO, Nairobi, Kenya

## Abstract

**Background:**

Many programmes on young people and HIV/AIDS prevention have focused on the in-school and channeled sexual and reproductive health messages through schools with limited activities for the young people's families. The assumption has been that parents in African families do not talk about sexual and reproductive health (SRH) with their children. These approach has had limited success because of failure to factor in the young person's family context, and the influence of parents. This paper explores parent-child communication about SRH in families, content, timing and reasons for their communication with their children aged 14-24 years in rural Tanzania.

**Methods:**

This study employed an ethnographic research design. Data collection involved eight weeks of participant observation, 17 focus group discussions and 46 in-depth interviews conducted with young people aged 14-24 years and parents of young people in this age group. Thematic analysis was conducted with the aid of NVIVO 7 software.

**Results:**

Parent-child communication about SRH happened in most families. The communication was mainly on same sex basis (mother-daughter and rarely father-son or father-daughter) and took the form of warnings, threats and physical discipline. Communication was triggered by seeing or hearing something a parent perceived negative and would not like their child to experience (such as a death attributable to HIV and unmarried young person's pregnancy). Although most young people were relaxed with their mothers than fathers, there is lack of trust as to what they can tell their parents for fear of punishment. Parents were limited as to what they could communicate about SRH because of lack of appropriate knowledge and cultural norms that restricted interactions between opposite sex.

**Conclusions:**

Due to the consequences of the HIV pandemic, parents are making attempts to communicate with their children about SRH. They are however, limited by cultural barriers, and lack of appropriate knowledge. With some skills training on communication and SRH, parents may be a natural avenue for channeling and reinforcing HIV/AIDS prevention messages to their children.

## Introduction

Sexual activity places young people in Tanzania at an increased risk of infection with Human Immunodeficiency syndrome virus (HIV), other sexually transmitted infections (STIs), as well as the potential for unplanned pregnancy [1]. As in many parts of sub-Saharan Africa (SSA), sexual activity begins early in Tanzania. By age 15, 11% of the girls and boys have had sex (ibid). In Tanzania, rates of condom use among young people are generally low [2]. In the 2003-04 Tanzania HIV/AIDS Indicator survey (THIS), although over half of the women and almost three quarters of young men knew where a person could get condoms, only 17% of young women and 26% of young men aged 15-24 mentioned they had used condoms the first time they had sexual intercourse [1]. What this implies is that although young people had some theoretical knowledge of HIV/AIDS and STIs, very few truly understand the risks around them.

The reasons why young people engage in sexual activity are complex and diverse and have been attributed to various social context and familial factors [3,4]. Contextual factors that increase or decrease susceptibility of young people to these outcomes include gender dynamics in relationships and within families, poverty, and cultural norms (ibid). Several interventions to promote sexual and reproductive health (SRH) have been developed and implemented, mainly targeting school-going young people in Tanzania [5]. A review of these interventions has shown that although they have had an impact on young people's knowledge about SRH, they have failed to change young people's sexual behavior [6]. The recent failures of school based interventions in Tanzania [6] and elsewhere [7,8] to show a positive impact on sexual behaviour may stimulate a focus on the wider socio-economic context that surround young people rather than exclusively on behavioural influences on individuals. As noted by other authors [9], determinants of sexual behaviour are a function not only of the individual but of structural and environmental factors as well. Therefore, a focus on families and particularly parents would provide support mechanisms for such interventions.

A large proportion of studies mainly from developed countries have been conducted on how parents influence adolescent sexual behaviour [10-14]. There is also a growing body of literature from sub-Saharan Africa [15-18] on the role of parents in young people's behaviour. Several programmes focused on the role of parenting in improving adolescent SRH have been implemented and experience from 30 of such programmes was described in a World Health Organization review [19]. In East Africa, there have been efforts towards exploring parent-child relationships and specifically parent-child communication. For example, in Uganda Kinsman [20] and Muyinda [21] have explored the use of traditional forms of socialization (i.e. the Senga), while programmes such as "straight talk campaign" have demonstrated the general willingness of parents and other adults to create a supportive environment for young people [16]. In Kenya, programmes such as "families matter" work directly with parents and their children to improve intra-familial communication about sexuality and sexual risk [17]. In Tanzania, Nyalali et al. [18] have examined general parent-child relationships and pointed to the strong social desirability biases inherent in questionnaires with parents about their relationships with their children.

Although there is overwhelming evidence [15] of the need to involve parents as part of the comprehensive strategy for improving young people's health and development, there has been conflicting findings on whether parents in SSA communicate with their children about SRH and on the effect of such communication on young people's sexual behaviour. While some studies [22,23] have shown that adolescents who discussed sex with parents were less likely to engage in unsafe sexual behaviours, other studies have not found a consistent relationship between parent-child communication and sexual risk behaviours [24,25]. These differences may be attributed to the content, timing and frequency of communication as well as the actual characteristics of parent-child relationships.

In SSA, a few studies have focused on young people's family interactions through parent-child communication about sex [22] and material support [26,27]. These studies have mainly focused on schooling young people with little consideration for the role of the out-of-school young people on the sexual decision making of those attending school. While the above studies have shown some effects, they are too scanty to be conclusive. They have recommended parental involvement in guiding adolescents in making responsible decisions around sex [26,27] without exploring if they actually currently do this, how they would do it and what exactly they should communicate and how and why they communicate.

Moreover, most of the studies focusing on communication about SRH in the developed countries [11,28] and SSA [22] have focused exclusively on secondary school going adolescents or those at tertiary levels [26]. This approach though relatively easy to execute, still omits out-of-school and primary school attending young people. As many of the East African countries still strive to achieve the millennium development goal (MDG) on universal primary education, only 39% of young people attend primary school and 23% reach secondary school [29]. In Tanzania, only 13% of children reach secondary school (ibid). Young people who attend school may be very untypical in terms of relative affluence, knowledge of HIV/AIDS and ways of thinking about the future. The present study explores if and how parents and other adult family members communicate with young people (in and out-of-school) about SRH (pubertal development, sexually transmitted infections (including HIV/AIDS), contraception, condoms, unplanned pregnancy, and any other sexual risks) focusing on the timing and reasons for the communication. Understanding communication patterns within the family makes it possible to better understand family connectedness, decision-making in the family, family regulations, gender role expectations and what is possible to communicate about SRH within a family context.

Most of the studies that have been conducted on parental influence on young people's sexual behaviour have collected information from young people and not their parents and other family members. This can result in information bias resulting in an unbalanced picture of what is actually happening in families and as regards parent-child relationships and communication about sex. This study involved both young people in and out-of-school and parents so as to gain a complete picture of their experience and a clear understanding of the family interactions and young people's sexual behaviour.

## Methods

To understand communication patterns in families and if and how parents communicate about SRH with their children, this analysis adopted an interpretivist approach emphasizing the importance of interpretation as well as observation in understanding the value and meaning that people ascribe to their behaviour [30]. Parents/carers of young people influence their sexual behaviour through interactions and communication of expectations. An understanding of this influence was gained through hearing the views of parents and young people.

Parents' own interpretations of expectations and interpretations of their children's sexual behaviour is considered crucial in determining what they communicate to them about SRH and what they expect others to communicate to their children concerning this. Therefore, detailed information about people's lives (from their own perspectives) and to some extent the researchers' own observations either of the circumstances in which they live or their engagement with research issues were crucial. Although the researchers' own interpretations are important, they are clearly delineated from those of the participants and therefore in developing the interpretations, the researchers adhered as closely as possible to the participants' accounts as the basis for interpretation.

Ethical approval for this study was provided by the Tanzanian Medical Research Co-ordination Committee. Additional permission to conduct the study was granted at the district, ward and the village levels (community and individual). In addition to seeking the consent of participants for those aged below 18 years (the age of majority in Tanzania), consent was also sought from the parents or caregivers. The purpose and methods for the study were explained to the potential participants, who provided verbal consent prior to participating.

### Design

This study employed an ethnographic research design. Data were collected using participant observation, in-depth interviews and focus group discussions. A combination of participant observation (PO), in-depth interviews (IDIs) and focus group discussions (FGDs) increased the understanding of complex issues related to family interactions, young people's sexual behaviour, and provided for a detailed understanding of parent-child communication. PO was purposely conducted prior to the IDIs and FGDs to help in the design of IDIs and FGDs topic guides by ensuring the questions in the guides were culturally relevant and appropriate, but also to clarify in IDIs and FGDs issues that had emerged during PO.

### Study setting and participants

The study was conducted in the Kisesa HIV cohort in North-Western Tanzania in a predominantly, Sukuma ethnic group [31]. The main religion was Christianity, while the main economic activity was farming. Data collection was conducted in 2007 and it involved 8 weeks of participant observation, 46 IDIs and 17 FGDs with young people aged 14-24 years old and parents/caregivers of young people of that age. Out of the 46 in-depth interviews, 25 were conducted with women (14 young women, 11 with female parents/caregivers) and 21 with men (12 young men, 9 male parents/caregivers). Eight of the FGDs were conducted with women (5 with young women, 3 female parents/caregivers) and 9 with men (6 young men, 3 male parent/caregivers).

Both male and female parents were included as participants as we were interested in understanding the interactions between parents and young people from both the parents as well as young people's point of view. Another reason for involving parents was because we were looking at parents in the light of potential SRH interventions as they are the main socialization agents in families.

### Procedure for data generation

Participant observation was carried out in one village by two researchers (1 male, 1 female). Prior to the start of data collection, the researchers introduced themselves in two public meetings held in the village. They lived in villagers' households and engaged with young people in their daily activities, in particular doing farm work and, for the women, collecting water and firewood and cooking. Young people in the host household and in contrasting households were befriended and accompanied to social events, such as markets, funerals, and video shows, and were informally interviewed. Most PO informants were young people aged 12 to 24 and parents/carers of young people within this age group. The researchers were encouraged to establish contacts with as representative a spread of young people in the village as possible, through the selection of their host families and by intentionally engaging with different groups and networks, e.g. religious and in and out-of-school. The researchers had greatest contact with their own sex, because it was not culturally appropriate to discuss sexual issues between the sexes. The male research assistant was from the Sukuma ethnic group and thus was able to follow participants' informal conversations easily. The fieldworkers wrote daily notes for one to two hours, and at the end of the field visit they wrote a summary report.

A snowballing approach was adopted for the selection of participants for the FGDs and this ensured that all the participants knew each other well and were free to discuss sensitive issues in each other's presence. Data were collected in two phases. During the first phase of the FGDs, three days were spent on getting to know and recruiting pre-existing friendship groups [32]. The FGDs were organised according to gender (male and female) and education status (in and out-of-school). The selection of participants for the second follow up phase of FGDs was based on a theoretical sampling approach [33]: two more FGDs with young women and men were conducted to explore further issues that had emerged from preliminary in-depth interview analysis.

Participants for IDIs were selected from FGD participants through purposive sampling (schooling status, sex, responses given during group discussion). The IDIs were held with a sample of the FGD participants so as to build on the rapport built during the group discussion and to explore at a personal level some of the issues that had emerged in the FGDs. This was to ensure that people whose views were relevant to the research questions were interviewed in detail. Theoretical sampling was used for the selection of participants for the seven follow up interviews. After reviewing data generated in the initial 39 interviews, the decision about who to involve in for follow up interviews was informed by the preliminary analysis, theory and emerging explanations from the initial data and three parents were interviewed a second time for a more detailed understanding of some of the issues they had raised. The other follow-up interviews were conducted with new participants to explore issues that had emerged in the first phase of the interviews.

### Analysis of data

The data were transcribed, translated to English, entered into QSR NVIVO 7 software and coded. A pragmatic approach to analysis was adopted whereby a combined use of an already designed coding scheme (anticipated codes) and grounded codes were utilized. Grounded codes were developed by a thorough reading of the data and they reflect the language and ways of expressing ideas as portrayed by the participants. The anticipated codes were developed from the research questions and repeated reading through of the data during the early stages of the analysis and refined in the light of further generation of the data. Thereafter, codes were later developed into more conceptual categories and finally themes. After the coding process had been completed, searches were carried out. The searching involved thoroughly reading the individual codes for emerging patterns. Theories were formulated and tested. An example of a theory is 'are parents who believed that premarital sex was unavoidable less likely to communicate messages discouraging sex?'. In order to answer this type of query, 'child codes' relating to parental beliefs about sex, nature of parent child communication, timing for the communication, and the motivations for the communications about SRH were searched. Thereafter, there was an attempt to explain the emerging patterns of associations e.g. why the observed patterns were occurring.

Although PO data provided background information on the activities and interactions in families and parent-child relationships, we have not directly used quotations from the PO notes in the paper. This is because the three methods used supplemented each other and hence we opted for more direct illustrative examples from the IDIs and FGDs where relevant but referred to a finding that resulted from PO where necessary.

## Findings and discussions

### Nature of parent-child initiated communication about SRH

Generally, some communication about sexual health was observed in most families. This communication was usually initiated by parents and rarely by young people and was characterized by warnings or threats. The topics for discussion were mainly about abstinence, unplanned pregnancy and HIV/AIDS. These communications reflected the worries parents had about their children's sexual health. However, among the issues that were rarely discussed in families were measures such as contraception and condoms.

#### Teaching expected behaviour through teasing and jokes

There was a difference between parent-child communication about SRH and this varied with tone, message and seriousness of what was being communicated. While parent-child communication was characterized by threats and warnings, that with grandparents were usually humorous. Most of the young people who lived with grandparents reported that they were closer to them than to their fathers. Their conversations were usually delivered as jokes and rarely as straight forward warnings the way parents did. Some of the jokes involved issues such as grandmother referring to her grandson as 'husband' while grandfather referring to granddaughter as 'wife'. However, these jokes did not imply sexual contact.

On the part of grandparents, they reported that they were comfortable talking about sex with their grandchildren and not their own children because they were not their children and hence there was nothing for them to fear. A grandfather living with his granddaughter said:

*She is my granddaughter and not my child. So I can't feel shy to talk about HIV...I don't tell her to use those protection (condoms), that is not my duty... the major thing is to prevent yourself against diseases *[IDI # 5, 71 year old male parent].

It was observed that grandparents were not restricted in what they communicated with their male or female grandchildren and hence were not concerned about being careful with what they said. The cultural norms around communication about sex across generations seemed to be flexible with them. This flexibility could be attributed to the traditional role observed in many African cultures where grandparents were the main sex socialization agents for grandchildren [3]. It is however, noteworthy to mention that although grandparents were comfortable discussing about sex with their grandchildren, they had limited knowledge concerning HIV/AIDS prevention, modern contraception and condoms and thus were limited in what they could communicate. They were also reluctant to discuss condoms and contraceptive options.

#### Masculinity and femininity

The parents' communication about sex further reinforced the societal expectations about masculinity and femininity. They linked femininity with abstinence until marriage while associated masculinity with sexual prowess. For example, the out-of-school young men mentioned that when their fathers were in a good mood, they sometimes talked about their sexual prowess with their sons:

R1: There is a day you are seated at home as a family, all happy. Then may be jokingly you talk to each other...Father jokes about how he used to attract girls when he was young...it is possible that the old man (father) has not seen you with a girl. He wants to assess your 'sharpness'. That I have narrated to you, it is now upon you

Rs: Laughter

*R2: Or you may find that some fathers until now they love women...so his aim is to lure you *[FGD #16, out-of-school young men].

On the contrary, mothers related femininity with sexual innocence. While seated with their daughters, they talked about how they had abstained until marriage and that they expected their daughters to do the same. Discussions such as this though intended to encourage young people to behave in ways that are in agreement with stipulated masculinity and femininity, they encouraged sexual activity among young men while reinforcing further the subordination of women. It is clear that some fathers were not good role models. They lured their sons into sexual activity by talking about their own sexual experience when they were young in a heroic way. Young men also talked about male relatives sometimes teasing them about sexual issues. If children are aware that their parents and adult siblings are having extramarital relationships, then they may not listen to their advice especially if it is on abstinence.

#### Communication about pregnancy

Parents did not seem to communicate with their primary school daughters about SRH issues with the same emphasis they did with those in secondary school. The communication was always delivered as general warnings and the only time it was specific and directed was when talking about the consequences of premarital sex on their education. Focusing communication to the secondary school daughters than the primary school was partly because they didn't expect those in primary school to be sexually active but also because of the high costs for taking a child to secondary school and did not want to lose them when the girl dropped out-of-school due to pregnancy. A father with a daughter in secondary school said:

*I told her, 'because you are going to school, you should be careful. There are unplanned pregnancies...therefore if you will have unclear issues (have sex) you will stop concentrating on what we took you to school for' *[IDI # 41, 42 year old father].

Although fathers were not close to their daughters, they expected their wives to be. Among some of the SRH issues they expected mothers to talk to their daughters about were avoiding unplanned pregnancy and focusing on their education. On the part of mothers, very few communicated about pregnancy prevention explicitly prior to pregnancy happening. In warning their daughters, they sometimes talked about their own experiences when they were young and the 'losses' they got when they had unplanned pregnancy. This is illustrated in the following excerpt:

*If you happen to have sex, this is bad and that is what happened to me (mother)...you get pregnant, there is no time for abortion. I can't kill. You will have to take care of that pregnancy. You will be expelled from school *[IDI # 37, 35 year old married mother].

Notwithstanding, for a few parents, the expectations they had about their daughters' future as being in marriage are changing. While others still emphasize marriage, a few have shifted focus to education. For those who emphasize education, they encouraged their primary school daughters to work hard at school and not rely on men. A mother who mentioned that emphasized hard work for her daughters (aged 14 and 15) by telling them to focus on their own employment said that she also threatened to forcefully marry them off, if they had unplanned pregnancy. This is illustrated in the following excerpt:

*If you do not get someone to marry you, then you will look for a job because nowadays there are many jobs especially if you complete primary school...Here you are in my family. I don't want you to bring me your family. I don't want to hear that you are pregnant. We will not take care of you any more or beat you, but you will go to live with that boy (boyfriend)...You better just protect yourself until you complete school, then if you are seduced it is okay because you are already an adult *[IDI # 7, 35 year old mother].

Although some mothers mentioned to their daughters that they should protect themselves from unplanned pregnancies, they did not explain how. To most of these mothers, protecting oneself meant abstinence. The mothers also perceived the out-of-school age as the adult age and as such the right age to have sex. This is a very interesting criterion for defining the right age for sexual activity, partly because the earliest age girls completed primary school was 15 years but usually it was 17 years or older. This might also help explain the high rates of unplanned pregnancies among the out-of-school unmarried women. It is possible that these young women may have the same views as their parents, that they are now adults and that their sexual activities are not limited by being in school. As noted by other authors [3,34,35], there seems to be an expectation for children to abstain while still in school. Similarly to what was noted in the above studies, there is evidence from these findings here that parents devote much effort to ensure that school pupils abstain. These efforts ranged from communication through threats about the consequences of unplanned pregnancies to a few carefully reasoning with them about the benefits of education and what the future held for them.

However, it is important to note that some of the SRH messages delivered by mothers may have been misleading to their daughters. Although meant to scare their daughters from engaging in sex and hence prevent them from having unplanned pregnancies, they may have actually increased their risks for it. A message such as 'immediately you engage in sex you will become pregnant' can be interpreted as misleading especially for the girls who have never had sex and who have not had their first menses. If they had sex and didn't get pregnant, they would be likely to get confused and start thinking that they are infertile. In a society where children are valued, the girl may then be tempted to have sex with several men to see if she is actually fertile and in the course of her experiments, she may end up with the much feared HIV or unplanned pregnancy.

#### Parents beliefs about contraception and reproduction

Parents did not encourage their young people to use contraception. This was because most of them believed this to be bad even though they had not tried using it themselves. They had fears concerning the side effects accruing from contraceptive use and one of their main fears was that contraception causes infertility. Infertility is something that is highly frowned upon in this society and anything that was associated with it was avoided. Participants' beliefs about contraception would be partly due to their knowledge about how the methods worked.

Mothers had their own understanding of reproduction and reported that they knew how their daughters perceived it. They said that they had not discussed contraception with their daughters for fear of being perceived as anti-reproduction. When asked if mothers talked about contraception with their daughters, they said:

R1: Very rarely...But this contraception the majority don't want it...They say, let her just give birth until the eggs are finished

*R2: If you tell her (daughter) about contraception from there she goes to tell her friend, 'my parent wants me to stop giving birth, but I have decided to just continue because I don't know which child will take care of me when I am old' *[FGD # 4, female parents].

It can be argued that a mother's own experience with contraception seems to have been important in the position they took about them. Only one out of the 11 mothers interviewed said had used modern contraception and had a positive view about them. She had told her primary school daughters that she would assist them to get Depo-Provera when they completed school. She said that she mentioned this to her daughters as a motivation for them to abstain until they completed primary school. Similar to most parents, she was certain that once her daughters complete primary school they would engage in sex. The eldest daughter was 15 years and hoped to complete primary school the following year. This participant said that she had mentioned this to her daughter when warning her about pregnancy. Her daughter aged 15 was also one of the two among those interviewed who had reported that she had never had sex:

*You will go to hospital or one day I will take you there because I have female friends who work there...they can instruct you on how to get family planning injections *[IDI # 7, 35 year old mother].

It is possible that this mother considered taking her daughter to obtain contraception at the local dispensary because she had used them herself and some of the staff were her friends. Therefore access did not appear to be a barrier for her the way it may have been for other parents and young women.

#### Communication about HIV/AIDS and STIs

This was the commonly discussed SRH issue in families. All participants had mentioned this as one of their major worries. Therefore, even parents who said had never talked about other SRH issues and neither had any plans of doing so with their children, mentioned that HIV/AIDS was the only thing they had talked about and would continue to talk about. This was because HIV/AIDS was considered a shameful catastrophe, and also one that interfered with the family economic resources and the family lineage through early deaths before young people were able to have families.

A male parent who had talked about HIV/AIDS with his children said:

*I talked about AIDS because once you begin suffering from it, the family economy goes down because you will concentrate on nursing the patient, so you can't even do your agricultural activities *[IDI # 32, 42 year old male parent].

Another male parent who perceived dying from HIV as very shameful to one's family as it indicated that one had been promiscuous said:

*For instance when you get this disease, it is normally very shameful to yourself and to your family because it seems like so and so's family or daughter was a prostitute *[IDI # 31, 42 year old father].

Young people's views about the severity of HIV/AIDS and hence the importance of talking about it were similar to their parents'. They reported that they thought their parents discussed HIV/AIDS with them because they loved them and had hopes about the future because of them.

In their discussions about HIV/AIDS with their children, parents reported used examples of some of their relatives who had died of AIDS to inflict fear in their young people about the dangers of sex and severity of the disease.

This is illustrated in the following excerpt:

*During that period when their uncle was sick from AIDS, I was telling them, 'let us go and see how your other uncles are suffering' (providing care). Then I told them, 'you have now witnessed, what is you opinion?' *[IDI # 29, 34 year old married woman].

Parents were aware about their children's vulnerability to HIV. Some of the few mothers who acknowledged that their daughters may engage in sex secretly without their knowledge, reported advising them to go for HIV test with their partners before they engaged in sex. In a group discussion with primary school girls they reported that they had been advised by their mothers to insist on testing before having sex:

*Some mothers say that it is better if someone loves you...seduces you, to tell him you should go for a test *[FGD # 8, primary school girls].

This was an interesting point of view because HIV test centers were not common in the study setting and moreover, stigma about HIV/AIDS is still common [36]. Given the group discussion dynamics, it is possible that these young people presented what they thought was acceptable among their peers and the wider community.

According to some parents, when they warned their children about the dangers of HIV/AIDS, they expected them to understand that this referred to overall SRH. Parents expected their children to think about what they had told them about HIV and to protect themselves. This was apparent in the discussion about sex which seemed to be very general and did not talk about specific SRH issues and prevention strategies. A male parent who felt warning his children about the dangers of AIDS was enough and meant he was referring to all the other SRH problems said:

*If you have already warned them about AIDS, they will just know that nowadays they must be careful with sexual matters. They have to meditate on that message themselves, they are adults *[IDI # 5, 71 year old father].

Parents lack of communication on specific SRH issues but assuming that the mention of the dangers of HIV/AIDS would mean everything, could be attributed to the consequences of shame and shyness of talking about other sexual health issues. It could also be as a result of their perception of the severity of HIV/AIDS in relation to other SRH issues. This may also mean that although parents may claim to be communicating about SRH with their children, they may not be doing this adequately.

Concerning SRH problems like STIs, a parent's own upbringing seemed to have been an important factor in their belief about what they felt was appropriate to communicate with their children. They drew on their experiences about what they had done when they had these problems and expected their children to do the same. Some male parents mentioned that they feared their fathers and could not talk to them about an STI infection. They interpreted telling their parents that they had an STI as being equivalent to telling them they were promiscuous. One of the male parents talked about his decision to talk to his friends instead of his father when he was young and had an STI in the following:

*You know that is a shameful problem to direct to your mother or father...It was not easy because you could be ranted at a lot...s/he may tell you, 'so you have started prostitution', so I had to hide it like that *[IDI # 32, 42 year old male parent].

#### Communication about condoms

Condoms were among some of the issues that parents mentioned outright were difficult for them to talk about with their children. The biggest dilemma for most parents seemed to be when to talk about different issues relating to SRH with their children. An overwhelming majority of participants felt that a parent or any adult talking to a child about condoms would be encouraging them to engage in sex which would contradict their messages on abstinence. Therefore, for the parents who strictly insisted on abstinence, discussing condoms with their children was not something they considered. In addition, some felt that they could not talk about condoms because they did not know whether their children were sexually active. Parental expectations for young people to admit that they were now sexually active for them to deliver the right advice (e.g. condom use) is a contradiction in a society where sexual activity was secretive and expected to be so [3,32]. Parents talked about condoms in the following:

*If I tell them to use condoms it is like allowing them to do such things because they will know that they will not get HIV or pregnancy...So I just teach them not to engage in those things (sex) *[IDI # 36, 56 year old married woman].

*Because she (16 year old daughter) said that they have never had sex. Now if you tell her you can disturb her mind, that 'this mother talks about condoms, does she want me to go with men (have sex)?' *[IDI # 30, 37 year old mother].

In addition to the widely held views that parents' discussion on condoms with their children was encouraging sexual activity, other barriers were: shyness and parents lacking appropriate knowledge about condoms. The majority of the parents (especially females) said that they had never seen condoms and thought they were not available in their village.

When mothers were asked if they could consider giving their sons condoms, most said that that was impossible. They wondered how they could do this and what that could mean to their sons. However, a few hypothetically talked about how this would work suppose they were required to do so in the following:

R1: if you bring them to your son you will be teaching him to go and have sex

R2: You don't just give him

R3: It is impossible

*R4: In fact it is just difficult to tell him...perhaps if they sleep in a separate house from yours [parent's], you just go and put on the table in their house where they sleep *[FGD # 4 female parents].

The way the parents in the above excerpt talked about the difficulties they would encounter if they were to give their sons condoms, shows the shame attached to mother-son communication about condoms. Notably, parents talked about this possibility in relation to sons and not daughters. This may be a manifestation of some of the beliefs they held about men as the ones who had control in sexual relationships.

Moreover, parental confidence in the effectiveness of condoms is important in determining what they talked about them with their children. Some parents said that they believed that condoms were not effective in preventing HIV and hence they did not see the need to talk about them. A 71 year old father was appalled to be asked if he talked to his children about condoms:

*Personally, I am quite against that [condoms]...I oppose that very much. That is not even something to discuss with people. Because condoms according to what they explain professionally only a very little percentage of people can survive...But the majority get infected, then secondly you build a strong base to make people to have sex more and more *[IDI # 5, 71 year old male parent].

The parents in the above excerpts argued against condoms. They lack trust in their effectiveness and perceive them to increase HIV risk through encouraging sexual activities. This clearly presents a barrier to the promotion of condom use in this community.

#### 'We live in a dangerous period'-Reasons for parent-child communication about condoms

Only one male parent out of the nine interviewed, said that he had talked to his children about condoms. This happened when he went for a trachoma seminar and he was given a packet of condoms. When he arrived home his children asked him about the packet. It was in response to this question that he got an opportunity to explain to them about condoms. It is noteworthy to mention that the seminar attended by this parent though not focused on sexuality, seemed to have helped him find a reason for communicating about condoms with his children. The same parent suggested that condoms should be made accessible to villagers. He had two daughters in secondary school and when asked if they were sexually active, he said that he thought they were not.

For a few of the fathers/uncles who believed that women were promiscuous and should not be trusted, advised their sons/nephews to use condoms. However, among the young men who mentioned that their father had talked to them about sex, only two mentioned that they had been advised to use condoms if they could not abstain. They said that they were cautioned by their fathers and sometimes uncles about getting STIs if they had unprotected sex. They were also cautioned about trusting girls because they were reputed to have multiple partners. It is noteworthy to mention that these were unique cases where parents/uncles openly talked about options such as condom use for those who failed to abstain. Even admitting that they would fail to abstain even after being advised to was not common.

A school boy said:

*Father advised me to stop that behaviour [having sex] completely. 'If it happens that you love a girl, then use a condom because we live in a dangerous period... there are so many diseases through sex at present compared to when we were youth (father) *[IDI # 21, 19 year old primary school boy].

The above excerpt points to what most of the parents presented about their beliefs about young people's SRH. Some parents believed that their children were living in 'bad weather conditions' (risky era). However, despite this awareness, very few parents provided practical prevention strategies for SRH problems facing their children.

Although there have been several SRH intervention activities working in the study communities for several years [31], it is worth highlighting that most of the participants had a vague idea about condoms. Most reported that they had never seen them and did not know where to get them. When asked about whether they would like to talk to their children about condoms in future, most parents said that they wondered how they could start such a discussion. Misconceptions and mistrust about condoms were also widespread as illustrated in the above excerpts. Parents have clearly shown their doubts about condom effectiveness. There biggest fear was about encouraging sexual activity if they talked to their children about this option. This highlights the importance of first understanding the parental views about SRH including preventions such as condoms before they are advocated for. To solicit for parental support in advocating for their children to use condoms, it is obvious that the negative attitudes are changed and misinformation corrected first.

#### Triggers and timing of communication

Communication about sex was spontaneous and was often triggered by: radio programmes, occurrence of a villager's death linked to HIV/AIDS, children coming home with flyers from school, parent perceiving a child's behaviour as risky, and when they saw a very thin/slim person they perceived was HIV positive. Examples of things that parents perceived as cues to being sexually active were being found chatting with a potential sexual partner, returning home late, befriending peers parents disapproved of their sexual behaviour and a child sneaking out or discretely inviting home sexual partners during the night. The communication was mainly in one direction with the parent delivering the warning and the young people expected to listen and heed advice.

As noted during participant observation, for the parent-child communications that were started by radio programmes, it usually happened in the evenings after dinner or after lunch. This was when most of the family members had assembled for a meal. The timing for the SRH radio programmes were usually in the evenings. It was observed that the presence of a visitor in the family provided a good opportunity for the discussion about SRH between parents and their children. Participants mentioned that this was because some adult visitors did not fear talking about SRH (e.g. pregnancy) because their own children were not present. Communications about sex in the family sometimes took the form of gossips (e.g. about an unmarried girl's pregnancy or rumours about someone being HIV positive) and was usually among same sex siblings.

There was a unique case of a mother who physically inspected her daughter's private parts as one of the ways of monitoring her sexual behaviour. This mother combined physical inspection with discussion about sex only when she was forced to, and this was when she heard from her friends that her daughter was about to engage in sex:

*There is a day I took my daughter with me to the farm... I asked her, I guess you understand that programme on AIDS in the world. I think you understand it, she said 'yes'. 'Whenever they talk you should be listening because there are some important teachings'. I then asked her, 'have you had your first menses?' she said 'no'...I then asked her 'Do you have a mchumba [sexual partner equivalent to a fiancée]?' When she kept quiet, I asked her 'how comes we are talking and you are quiet? Talk if you have a mchumba'. She remained quiet. Then I told her, 'why are you quiet, so you have started involving in sexual activities' *[IDI # 14, 34 year old mother].

This excerpt is an example of the form of communication mothers employed to investigate about their schooling daughter's sexual activity. This mother decided to talk about sex after hearing from a neighbour that her daughter was about to have sex. Unfortunately, by the time the mother initiated the discussion, the daughter had already had sex. Although she had secretly employed monitoring techniques involving touching in her daughter's private parts while she was asleep, this had not prevented her daughter from engaging in sex. It is evident that timing for parent-child communication about SRH has to be done early. Waiting for clues that a child has started sex before initiating such discussion may be too late. It is clear that this mother's monitoring techniques involving physical inspection of her daughter's private parts did not seem to have worked to prevent her daughter from resisting pressure for engaging in sex. This mother had not provided enough and appropriate advice about what her daughter should do in case she was faced by unanticipated challenges such as forced sex. She had also never talked about protection and hence when her daughter was pressured to have sex, she had unprotected sex. Moreover, due to the lack of closeness with her daughter and the cultural expectations of secrets and silences around sex [34,37], when this happened her daughter did not inform any family member for fear of punishment.

SRH materials given to children at school were very helpful starters for a parent-child discussion on sex. A father whose communication with his daughter had been facilitated by a flier said:

*For instance on the day when I saw her with those fliers, I told her 'stay away from sex'...I started the discussion after seeing the flier *[IDI 31, 42 year old male parent].

Triggers have clearly emerged as very important for starting parent-child discussion about sexuality. For the families which had never had a trigger, starting a father-daughter SRH conversation was particularly difficult and some fathers mentioned this as the reason for their lack of discussion. However, they did not have a plan of doing this. A male parent talked about this in the following:

*I don't even intend to [talk about sex]...You can't just begin telling her unless there is a conversation that leads you to that stage...I think there has been nothing to make us talk about it and that is why I have never talked about it with her *[IDI # 32, 42 year old male parent].

#### The right time for communication

Parents mainly communicated with their children after observing changes in their behaviours which they attributed to them having sex. However, most of the parents were in agreement concerning the fact that young people have to be advised about sex before sexual debut. They said that once someone has started having sex, it was difficult to stop them. When they were asked the age they thought was right to talk to their children about sex, most said 13 years for the girls and 15 for the boys. As noted in these findings, the biggest challenge seems to be how to start the discussion about sex with one's children without a reason.

*After a parent discovers that s/he has a sexual partner that is when it is possible to feel free to tell her now *[IDI # 3, key informant/male parent].

Although some parents mentioned that parent-child communication about SRH should start at an early age, others believed that advising their children about sex at a very early age may not be very helpful as they may not understand. A mother who said had warned her daughter about engaging in sex at the age of 13, had not done the same for her son because she perceived him to be small, reported:

*No, they [sons] are still small...the eldest is 13 years....now when his age comes to 15 years at least he will have matured and more reasonable to counsel. But now it is not easy for them to understand anything *[IDI # 14, 35 year old mother].

Moreover, parents who were against advising children about SRH before they saw signs that they were sexually active had concerns about spoiling (teaching) their children to engage in sex if they talked to them about sex at an early age. A male parent talked about this in the following:

*Now if you start engaging them in issues like those [discussion about sex], sometimes we fear that they may get spoiled commence sexual activity] *[IDI # 41, 42 year old father].

The timing of parent-child communication about sex seems very important in this setting. While parents may wait for clues that their child is sexually active before they initiate a discussion, it may be too late. Waiting for clues may also be a difficult thing because of the secretive nature of sexual relationships. Moreover, this may mean that very secretive young people may be at increased risk of SRH problems as they may not get timely advice/warnings on prevention.

### Frequency of communication about SRH in the family

As much as parent-child communication about sex is important, equally important is the frequency of communication. Since the discussions about sex in this setting depended on something happening, it was difficult for most of the participants to tell how often they did this. For the few who were able to estimate this reported that the frequency of communication in their families ranged from once in a day to once in a month or several months.

Most of the male parents felt that talking about sex with one's children was not a pleasant thing and hence they had to be an important reason for them to do it. When asked about how frequently he talked about SRH with his children, a 42 year old father said:

*It is not very often...Like I have said, normally parents don't want to talk about it sex]...let's say its like there is no need...Or if there is nothing leading to it, so to say let's talk about this...I mean there must be an issue, the issue alerts him/her that there is this and this...now you use, that issue *[IDI # 31, 42 year old male parent].

Some parents felt that the frequency of discussion about sex with their children did not matter much in terms of child sexual behaviour. They reported that what mattered was the child's ability to adhere to advice. A 34 year old mother who believed in this said:

*If a child is obedient, s/he will just obey...but if you kept repeating and s/he is not obedient it is not helpful *[IDI # 20, 34 year old mother].

### Perceptions of parents on parent-child communication about SRH

It was observed that both parents and young people used euphemisms to refer to sexual issues during discussions with researchers. It was also noted during participant observation that most parents were careful in their selection of words when giving warnings related to sex to their children. At the beginning of the discussion about SRH, participants were told to mention some of the words they used to refer to sex, they mentioned words such as 'act',' act of marriage', and 'meet bodily'. However, during the actual discussions, they struggled to look for appropriate terminology and avoided using explicit sexual terminology. Their discomfort about explicitly mentioning sexual terms shows how difficult it was for these parents to talk about sex with their young people. This difficulty could be attributed to the sexual norms in this setting that prohibit openness about sex across genders and generations.

When asked about how they felt talking about sex with their children, most of the male parents said that they perceived talking about sex with one's children as shameful, immoral and encouraging the child to have sex. Some male parents said outright that they did not discuss SRH with their children because there wasn't anything for them to discuss other than just warning them. Warnings were delivered whenever they noticed that their children were behaving in unacceptable manner. They also did not perceive the warnings to be a discussion about SRH. One of the male parents who shared this view said:

*This does not take time for me to talk [about sex] with them. I just say outright that time has come for each one of you to be careful...'Do you see the deceased young man, he died from AIDS, so you must be very careful with these areas of ours, they are full of AIDS'...You finish the lesson, there is no discussion, what will you discuss there?...you are just giving them a warning *[IDI # 5, 71 year old man].

During focus group discussions, most of the parents reported that they thought other people's children were already sexually active. However, in the individual interviews, most said that they thought their children were not sexually active but maintained that other people's children were. Coupled with feelings of shame, perception of their children as not being vulnerable may have been a barrier to a parent starting a discussion with their children. A male parent who attributed the lack of communication to parental shyness said:

*To tell a child before you discover that s/he has engaged in sex, perhaps most parents find it shameful *[IDI # 3, key informant].

Participants mentioned that a parent's ability to talk about SRH with their children was influenced by parent's level of education and concern about their children's future and the dangers of HIV/AIDS. They said that they think those who had never attended school found it more difficult than those who had some formal education. This argument seems plausible given that education may have enhanced their knowledge on SRH. It is also likely that it reduced the barrier that made it difficult to discuss sexuality as their outlook towards health broadened. Mothers talked about the difficulty of discussing SRH in the following quote:

*It's very rare, perhaps you may get two out of ten...I think it is one who has got education or she is serious with her child's future because if her child gets HIV it will be a burden to him/her *[FGD # 4, female parents].

Young peoples' views concerning sexual issues being confidential was also in agreement with what some male parents reported concerning discussing their children's SRH issues.

Some male parents said that they did not talk to their children about SRH because they perceived sexual issues to be private. A male parent who had not talked to his children about SRH for this reason said:

*I have never, you know it is not easy to discuss [sex] with her because those are confidential things...She can't tell you and I can't even ask her... I don't want to talk about it completely...it is really shameful to talk about such things with your children *[IDI # 32, 42 year old father].

When asked to explain what he considered as confidential the parent in the above excerpt said:

*Confidential things are things that are private to her and she doesn't want to disclose...Now she can't begin to tell you that I have a partner... It's impossible, that is her secret *[IDI # 32, 42 year old father].

It is evident from this excerpts that some parents held strong beliefs that parents should not discuss sexuality with their children. They felt that sexual issues are secret/confidential issues and not issues to be shared with one's parents. They were clear about not wanting to discuss these issues with their children. It is interesting to note how parents selectively applied respect for confidentiality. While most of them had low confidence in their children's behaviours, generally, they mentioned the lack of communication about SRH to respect for privacy. This in a way was selectively applied to justify their lack of communication about sex with their children. Other researchers working in different contexts have found similar findings [34,37]. They noted that parents rarely discussed aspects of their young people's sexual development. For example, Lesch & Kruger [34] noted that mothers were not only reluctant to communicate verbally with their children about sex, they also tended to discourage such communication through non-verbal messages.

Concerning communication about SRH with sons, some parents reported that they had not talked to their sons about this because they were not close and therefore talking to them would be a waste of ideas. A single father who felt that the emotional bond between him and his son was weak and hence discussing SRH with him was useless said:

*You see someone just like that, he [son]can't come close to you so that you may discuss...now you also just ignore him...If you tell him something as his father, he wouldn't care, now ... days go by and we continue ignoring each other...Other people's sons talk when they are eating but not my son. When he finishes eating he washes his hands and disappears. Now what discussion can you have with such a person? ....That will be troubling myself. I will be wasting my ideas *[IDI #2, 60 year old father].

The single father in the above example sounds bitter about the behaviour of his son. He is not willing to discuss SRH with him and perceives doing that as a waste of ideas. It is clear that this parent did not trust his son. It was observed during PO and clarified in the IDI that the son to this single father helped to support the family and during an interview with him, he had shown contempt for his father because he was an alcoholic and rarely provided for their family. While the son felt that his father was irresponsible and an alcoholic, the father felt that his son did not value him as seen in him not spending time with him and not listening to his advice.

In a follow up discussion with the single father in the above example, it was noted that although he felt his 17 year old primary school daughter was better behaved, than the 24 year old son [in the example above], he had not talked to her about SRH. This father like many others perceives his daughter accepting each and everything he tells her as respect. He feels closer to his daughter because she is obedient and never questions his advice. However, he could not discuss SRH issues with her because he did not know how to do this. The only sexual issue he was willing to discuss with his daughter however, was her marriage proposal. When asked if he had talked about sex with the daughter he said:

*What would I discuss with her about?...may be if someone will come to engage her, then we will discuss...because how will you discuss such things while she is just alone....How will I begin that to a child? *[IDI #2, 60 year old single father].

As can be seen from the above excerpt, this parent is one of the many who found it difficult to discuss SRH issues with their children. He blamed lack of discussion with his son to the son's behaviour. However, although he had mentioned that he loved and was close to his daughter, it emerged that he was uncomfortable talking about SRH with his children unless it was about marriage.

Parents perceived the closeness they had with their sons and daughters differently. Fathers reported that they thought they were supposed to be close to their sons and not their daughters and thus argued that they could not talk to their daughters explicitly about SRH because this was a taboo in the Sukuma traditions. They however said they did this through their wives. When asked why fathers thought that mothers were the ones supposed to talk to daughters in details about SRH and not them, one of the fathers said:

*You know between a mother and a father, the female child is closer to her mother and not her father...when it is a boy, he is closer to me *[IDI # 23, 44 year old male parent].

The examples point to fathers giving different excuses for lack of discussion about SRH with their children. Although they reported that fathers should be close to sons while mothers to daughters, this was not the case.

In spite of fathers being aware of what was required to enhance closeness with their children, they respected traditions more and as a result maintained distance. They expected their children to fear them the way they too had feared their own fathers. A male parent talked about his experience in the following:

*You know as a child, I was very close to my grandfather...so when I had a problem I feared that perhaps if I tell father, he can slap me...You know in Sukuma traditions, you have to fear your father...but with grandfather, he keeps calling you 'grandfather too', it brings your relationship with grandfather closer than with father *[IDI # 32, 42 year old male parent].

### Perspectives of young people on parent-child communication about sex

#### 'Only parents with love chat with their children'-Lack of parent-child closeness and communication about sex

Parent-child closeness referred to the emotional distance that existed between parents and their young people. It was manifested in whom and how young people felt free to interact with and confide in when they had a social need. Social needs referred to the non-tangible needs (emotional) that young people had. For example, need for advice concerning sexual relationships and handling of SRH incidences (such as unplanned pregnancy, and having STIs). The feeling of parent-child closeness was very important in determining parent-child relationship and communication about SRH. In general, parent-child closeness was low.

Parents' expression of love to children of the opposite sex through informal chats and spending time together were rare. They were limited by parents' personality as well as the cultural norms that stipulated appropriate relationships between family members of the opposite sex. These norms encouraged fear as appropriate behaviour. Young people from families with both parents reported that they were happier in their mothers' presence than they were with their fathers'. Young women interpreted the silences that existed between them and their fathers as their fathers perceiving them as having nothing important to tell them. On the contrary, mothers were mentioned as the most caring and loving.

Young people talked about this in the following quote:

*Only parents with love chat with their children [FGD #8, primary school girls]*.

Regarding father-child closeness they said:

I: Is there a time when fathers chat with their children?

R1: It is very rare

*R2: May be if he has a good heart, he is happy with his children...now if you find that father has a bad heart, he has no love for his children...he does not want to talk to his children, both the males or females. I mean that love is lacking*.

R3: ....*You find that to sit with him at home after an evening meal...you start to discuss a certain issue, it is very rare in most homes...in some cases it is totally absent...after an evening meal you go to sleep. When father completes eating he leaves for bed. Will you go to pull him out, 'father come we have a chat?' he does not even want to talk to you *[FGD # 17, out-of-school young women].

The above excerpts have illustrated the relationships that existed between fathers and their daughters.

#### Schooling status and child willingness to discuss SRH issues with the parent

Schooling status was an important determinant of young people's willingness to discuss SRH with their parents. While most of those attending school mentioned that they would like to discuss SRH with their parents (even though most had never done that), most of the out-of-school said that they would not. The school pupils who said had discussed sex with their parents said they were satisfied with the discussions because the parent was helping them to avoid SRH risks. They acknowledged that parental guidance and restrictions on their behaviour was important for their sexual health. They also mentioned that they trusted that their parents gave them the best advice and talked about the consequence of disobedience on one's family when a disobedient young person returned home for parental care with a serious health problem (e.g. HIV/AIDS).

Parental reaction to their older daughter's unwanted pregnancy was very important in determining how the younger siblings perceived their message on this. Some school girls said that although they feared pregnancy, they knew their parents could not do anything when it happened since they had not done anything to their older sisters when they had it. This is despite of all the threats they had given them prior to the occurrence of pregnancy. When asked about how her parents would react if she had unwanted pregnancy, a school girl said:

*They will not do anything....when my sister became pregnant they did not say anything *[IDI # 18, 17 year old school girl].

There were however, a few primary school girls who mentioned that they did not want their parent to talk to them about SRH issues because this would be teaching them. An example of a school girl who held this view said:

*If I talk to mother about such issues its like she is teaching me...that is why mother has no time for such issues *[IDI 26, 17 year old primary school girl].

#### 'I fear it because I have no where to go if I get pregnant'

Unwanted pregnancy was one of the big fears for the young women who had never had sex. Due to the constant parental warnings and threats about the consequences of sex, 2 out of the 14 young women interviewed reported had decided to abstain. One lived in a both parent family while the other lived with maternal grandparents. In an in-depth interview with the mother of the one from both parent family, she had mentioned that she usually talked to her daughter about the dangers of engaging in sex. When the girl was asked why she had decided to abstain, she said that she mainly feared pregnancy and STIs. This corresponds with what her mother had said about what she communicated with her. This may mean that the girl had internalized the message communicated to her about pregnancy. She understood that pregnancy was something to be feared and since sex was the only likely way to get it, she had to avoid it. When asked why she had never had sex, the girl said:

*Because there are so many diseases, and I will get pregnant...I fear it because I have no where to go if I get pregnant and also many people at home have high expectations in me *[IDI # 16, 15 year school girl].

It is clear that this girl equated having sex with getting pregnant and diseases. Her decision to abstain is mainly because of the fears and threats from her mother. Linking this interview with the one with her mother, it seems like most of what her mother communicated in warnings had had an impact on her sexual decision making. In the interview with her mother she had mentioned that if she had unwanted pregnancy, she should never come back home. These findings are consistent with what was observed in South Africa, [34] where mothers were noted to be powerful agents in the young women's constructions of their own sexuality. By presenting sex as a very dangerous activity to their daughters, mothers unintentionally contributed to their daughters' limited sense of sexual agency.

#### Parents' ability to provide practical advice

Young people's continued trust in their parents advice depended on how the parent had solved a previous SRH problem they had. They needed practical solutions and hence expected certain responses from their parents when they approached them with sexual health problems. Lack of satisfaction with a parent's response was a discouragement for the child to further confide in the parent when they had other problems. A 20 year old woman talked about her lack of satisfaction when she had a SRH problem in the following excerpt:

... *When I was menstruating a lot of blood, I only told mother but she did not tell me anything. She only said that, 'you appear to be sick. You are supposed to go to hospital and tell them'...I mean I was not satisfied with that [response] because when I told her, she should have told me that 'let me take you to the hospital', but instead I should go alone...Every time I start menstruating, I tell my mother but she still tells me I should go to hospital myself *[IDI #10, 20 year old *msimbe *woman].

Although young people may know where the health services are, they sometimes needed their parent's help to access them. This could be due to fear of the health workers, but also because they did not know how to explain their problem to the health personnel. It is possible that the parents may also be experiencing similar barriers to accessing SRH services as their young people. They may be shy accompanying their daughters to the hospital and helping them explain their problem. As seen in the above excerpt, the 20 year old young woman continued suffering without seeking health care because the person (mother) that she trusted to help her to find a solution did not.

#### Selective adherence to parental advice on SRH

When parents were asked about their views concerning the satisfaction of their young people with the advice/warnings they gave them about sex, most of them said that they assumed they were satisfied. They said the only way they could tell that they were not satisfied is if they had unwanted pregnancy or got infected with HIV. Hence parents who had out-of-school unmarried daughters who had not had unwanted pregnancy regarded themselves as successful in their parenting. They attributed their daughter not having had unwanted pregnancy as being satisfied with the advice they gave them about abstinence.

A young woman's decisions to end or start new relationships were sometimes influenced by their families' expectation of them. An example of such an expectation was through marriage. As part of the general family advice, most parents did not want their daughters to elope but insisted on the girl getting married formally. Some of the young women were satisfied with this advice and kept changing boyfriends instead of eloping with those who wanted them to. An example is a 20 year old woman whom although was not satisfied with the parental advice on abstinence, she heeded the one on not eloping. She believed that eloping was wrong and thus ended relationships with partners who wanted this. This is illustrated in the following excerpt:

*When I told him [boyfriend] to come home and engage me, he said that he did not have the financial ability. He wanted me to elope...And for me to elope, it is not normal...Because father told us that the traditions of that home are against that [eloping]. That is why I decided to leave him like that [first partner] and went with this one [second partner] who came home to engage me *[IDI # 44, 20 year old *msimbe *woman].

It is noteworthy to mention that young women selectively heed advice from parents. As can be seen from the above excerpt, this young woman followed the advice on not eloping but not on abstinence. She opted to get married to someone she did not know well because he came home to propose to her parents. Unfortunately, the marriage lasted for less than a year and she returned to her parent's home. She reported that she had revived the relationship with her old partner (who had wanted to elope with her). Since this man still wants to elope with her and she still feels it is not right, she said that she had decided to get a pregnancy out-of-wedlock so that her parents could allow her to get married to him. This is an example of an incidence where parental communication led to confusion and hence exposing the young person to risk. Deciding to have a pregnancy so that she could be allowed to marry the man of her choice, means this young woman had unprotected sex which further exposed her to the risk of HIV.

A few parents thought that their children were not satisfied with their advice as was seen in the child's behaviour such as not marrying when advised to. Other signs that parents mentioned as signs of child's lack of satisfaction with communication is when they disobeyed advice and continued engaging in sex.

## Discussion and conclusion

This analysis examined whether parent-child communication about SRH exists, the nature of content, timing and frequency and young people's satisfaction with the communication. Parents were questioned about the sexual behaviours they expected from their young people, their worries and if and how they communicate with them about sexual health. Our findings have indicated that discussions about SRH in families do happen and that communications were mainly about abstinence, HIV/AIDS, unwanted pregnancy, marriage and focus on education. It was observed that most of the communications were in favour of marriage as the end reward for 'good behaviours' and in cases where parents were tolerant about children's engagement in sex, encouraged them to have one partner. The communication was always initiated by parents and tended to focus more on the young women especially those still schooling. These findings point to the existence of parent-child communication about sex and their interest in the sexual health of their children.

The findings have demonstrated that parent-child communication about SRH was mainly delivered by mothers and rarely fathers. Mothers were considered close to their children and spent longer periods of time with them than fathers. A young person feeling close and cared for by their mothers was important for the mother-child communication about SRH. However, although mothers had the advantage of being trusted by their children, they did not fully exploit this for a friendly discussion about SRH with their children. Therefore, similar to the fathers who were considered as not close, they too communicated about SRH through threats and warnings. The mother-daughter difficulty communicating about sex observed in this study was also noted in South Africa [34] and Kenya [37]. Open and clear communication is crucial for passing on messages about SRH.

Although what parents communicate about SRH with their children is crucial, equally important is the timing for communication. Most parents waited for clues that a child was sexually active before they warned and threatened them about the consequences of engaging in sex. Parents communicating only after they realized that their children were sexually active, is likely to have had little impact on their protection use. This finding supports some of the recommendations in other studies about initiating discussions on sex with young people before sexual debut for a more desired SRH impact [5,38] and hence the motivations of some school based SRH interventions [5,39] to target SRH education to school children before they become sexually active. However, it is noteworthy to mention that there is dearth of literature from SSA on the effect of communication (e.g. quality, timing and frequency) on young people's sexual behaviour. As noted in some studies [13,28] aspects such as quality and frequency of communication are important in parent-child communication. This would probably help shed light on the lack of change in young people's sexual behaviour in this setting despite some occurrence of parent-child communication about the dangers of sex.

These findings point to the importance of other forms of SRH information on influencing parent-child communication about sexuality. Triggers from radio programmes and other sources have clearly emerged as good starters for such communication within the family. What this implies is that mass media programmes should be encouraged more and if possible such programmes should have specific tips on how parents should have meaningful discussions on sexuality with their children. Parents need skills training in areas of parent-child communication on SRH so that they are able to give appropriate, timely and non-contradictory information. Parent-child communication was hampered by the shame and fear surrounding sex as seen in the question most asked, *'How do I start the discussion'*. This is particularly for children of the opposite sex who may be so disadvantaged in single parent families. Therefore the taboo about cross-sex and cross-generation communication has to be carefully taken into consideration.

The implications of these findings are that the family and parents in particular, are important factors that should not be ignored in programmes that wish to reduce young people's risky sexual behaviours. Young people's SRH risk prevention programmes in Tanzania have usually been delivered in school contexts or directly to young people through the media and health workers. Although there is a considerable body of evidence (mainly from developed countries), on the importance of parents regarding young people's risky behaviours, [11,13-15,28] very few programmes in Tanzania have included parents or worked with them [18], let alone attempted to strengthen families for young people's good. These findings reinforce the need for developing programmes to support parents to stay involved in the lives of their young people (i.e. in both the in school or out-of-school groups) and to change their perspectives about their children's sexuality. Programmes should teach both parents and young people to communicate explicitly and clearly. Hence, sexual health prevention programmes that target both parents and young people regarding communication may be of greatest value on safe sex behaviors.

Although parents' primary goal in their communication about SRH was to discourage sexual activity among young women, in reality this did not deter young women from engaging in sex: most of the participants who were young people in our study reported that they were sexually active. In fact, it appears that such discouragements and lack of parental acknowledgement that unmarried young women were sexually active, led to enhanced secrecy in their sexual relationships. Although the secretive nature of the sexual relationships maintained some degree of harmony between the young person and other adult family members, it had implications for a young person's SRH. This was because it made it difficult for them to freely access protection (i.e. condoms and contraception) for fear of being discovered that they were sexually active. Moreover, secrecy encouraged opportunistic sexual encounters to flourish because young people seemed to take advantage of any opportunity they had, when their parents were not present, to have sex. Although secrecy in sexual relationships is culturally acceptable [3,40] too much secrecy is detrimental to young people's sexual health and has implications for interventions advocating openness between parents and young people. Our findings therefore raise important questions about how parents may perceive new ideas of openness to discuss SRH and the openness of their children to express their sexual feelings (through open relationships). In as much as parents thought they were protecting their children by not acknowledging their sexual activity and favouring the culture of secrecy in sexual relationships, they in actual fact might be increasing their risk for HIV/AIDS and unplanned pregnancy as this secrecy and opportunistic sexual encounters makes it difficult to plan for, and access condoms and contraception. Hence, parents acknowledging that their children may be sexually active may offer a good opportunity for young people to develop their sexual relationships and hopefully plan for the use of condoms and other contraception when they need them.

As much as parent-child communication about SRH is important, equally important is how the communication is conducted and perceived by the young person. The findings have demonstrated that young people valued practical advice to their sexual health problems. Most of the parent-child communication was also hampered by limited parental knowledge about HIV and other SRH matters. Young people appeared more knowledgeable about SRH issues (e.g. HIV and condoms) than parents. This concurs with what was noted in Uganda [20,21] where the traditional sex educators (known as *ssenga*), felt that their role was no longer valuable at the present time since young people knew more than the traditional *ssengas *(sex educators). Other studies point out that for familial support to influence young people's behaviours, they have to perceive such communication to be satisfying and congruent with their individual goals [28]. Whitaker *et al*, [28] noted that parent-adolescent communication about sex promoted responsible sexual behaviour only if parents are perceived by young people as skilled, comfortable and open in discussing sexuality. Therefore, young people who reported that their parents held skilled, open interactive discussions with them were significantly more likely than the young people of the less skilled communicators to use condoms at most recent intercourse and across time [13,41,42]. What this calls for is efforts to improve parental knowledge on SRH so that they can be able to communicate in an informed and convincing way to their children.

Among some of the issues that parents rarely talked about with their young people were the use of condoms and contraception. Parents' reluctance to talk to their children about these issues was because they believed that such discussion encouraged them to have sex which was culturally not acceptable. This belief is in keeping with what has been reported by other researchers in the SSA [3]. The above authors have also indicated that parents may be reluctant to allow their children to participate in sex education delivered through schools and other external sources because they believed that those who participate in sex education are likely to become prostitutes because such education encouraged options such as condom use and undermined morality. However, this view has conflicted with two studies which show that sex education does not cause promiscuity [24,43].

The main motivation for parent-child communication about SRH was because of the parents' fears concerning the dangers of HIV/AIDS among their children and efforts to ensure abstinence until marriage among their daughters. Although parents focused on abstinence messages, this is not an appropriate goal to aspire to in SRH education because in reality it was difficult to achieve. Rather the goal should be to develop young people who can exercise agency and are able to manage their own SRH. Parents need to be aware that scare tactics may work for a limited while and only for those who fear punishment from parents but do not facilitate self-reflexivity and internal locus of control regarding one's own sexuality. This might explain why most young women continued having sex despite parental threats and warnings.

Findings from this study show that lack of direct parent-child communication about sex has been attributed to lack of parent-child closeness, shame, fear and cultural norms. However, what was noted here was that most parents were forced to communicate through warnings. This method of communication can be attributed to the severity of the HIV pandemic where parents are feeling obliged to do something to save their children. Several authors have attributed the difficulty of parent-child communication about sexuality to the 'sex taboo' [3,24,34]. They argue that the sex taboo prohibits the discussion of sexual matters between people of different generations. This could offer an explanation for the limited communication about SRH experienced by parents in this study. However, the findings in this study point to the positive changes that are slowly taking place.

The most encouraging aspect from these findings is that progress is being made as seen in parents overcoming some of the traditional beliefs around communication about SRH and the expectations they had for their male and female children's sexuality. As mentioned by parents, in the past, their concerns and communication about sexual health were focused on abstinence and unplanned pregnancies for their daughters, but they are recognizing the need to also focus their communication on their sons' behaviours because of the consequences of HIV/AIDS. Similar to our findings, other studies have also noted parents focusing more communication about SRH especially those related to abstinence and pregnancy avoidance on the female children than the males. As demonstrated in the findings, HIV/AIDS was one of the few issues that parents unanimously agreed about - that their sons' SRH was also at risk - and hence should be a target for SRH education as well. Therefore in as much as parents would like their sons to prove their masculinity (by having sex), they are now coming to terms with the dangers of HIV/AIDS. We argue that HIV/AIDS is gradually changing the dynamics of the traditional beliefs parents hold about their male and female children's sexuality and the taboos around communication about sexuality. Parents seem to be learning the hard way by seeing the consequences of HIV/AIDS in their communities and there seems to be hope as seen in their willingness to want to prevent their children from infection. We believe this willingness for change may provide a good avenue for interventions to focus on parents as one of the channels for SRH information delivery to young people and to solicit their support on issues related to young people's risk.

Notwithstanding our confidence in the findings, this study has its limitations. As most of the data were collected in a qualitative way, it is not possible to quantify parent-child communication and specifically, characterize this by parent's and child's sex, but also to attribute those elements of communication that had an effect on sexual behaviour. A follow up questionnaire based study could provide some insights in these areas but would lack the capacity to capture the nuances of communication seen in the present study. Despite its limitations, this study demonstrates that parent-child communication do occur in families and that it is possible to channel SRH interventions focused at young people through parents and the wider family. This would provide a useful approach in addition to the existing efforts, to stem HIV/AIDS and other SRH problems among young people. Moreover, involvement of family members, particularly parents, would encourage parental approval and support to sexual health promotion efforts from external sources and channeled through other avenues such as schools, health facilities and media.

## Competing interests

The authors declare that they have no competing interests.

## Authors' contributions

JW designed the study, conducted the field work, qualitative data analysis and drafted the manuscript. AF participated in the analysis and writing of the manuscript. MU participated in the write up. BZ participated in the write up. WS participated in the design of the study and write-up. All authors read and approved the final manuscript.
